# Retrospective study of the reproductive performance of Barb and Thoroughbred stallions in Algeria

**DOI:** 10.14202/vetworld.2019.1132-1139

**Published:** 2019-07-26

**Authors:** Nedjma Aouane, Abdelkrim Nasri, Mohamed Al Amine Bekara, Ahmed Khireddine Metref, Rachid Kaidi

**Affiliations:** 1 High National Veterinary School of Algiers, Oued Smar, Algiers, Algeria; 2 National Stud farm of Chaouchaoua, Tiaret, Algeria; 3 Laboratory of Molecular Biology, Genomics and Bioinformatics, Department of Biology, Faculty of Nature and Life Sciences, University Hassiba Benbouali of Chlef, Algeria; 4 Laboratory of Biotechnology of reproduction, Veterinary Science Institute, University of Blida, Algeria

**Keywords:** age, Barb, breeding performance, breed, stallion

## Abstract

**Background and Aim:**

The Barb horse occupies a prominent place in the history, culture, and equestrian traditions of the Maghreb (Algeria, Morocco, and Tunisia). Although many studies on the breed standard and morphometry have been published, there are no studies on its fertility and reproductive potential. Thus, this work aimed to study the fertility of Barb, Arabian Purebred, and Thoroughbred horses in Algeria.

**Materials and Methods:**

A total of 168 stallions and 1202 mares at the Chaouchaoua Stud farm in Tiaret, Algeria, were included in the study. The reproductive performance during 1592 cycles over 10 consecutive mating seasons (2003-2012) was evaluated. Multivariate analysis with logistic regression was used to analyze the reproductive parameters such as the number of cycles operated, number of pregnant mares, pregnancy rate per cycle, seasonal pregnancy rate, and embryonic mortality rate, and to determine the influence of breed, stallion’s age, and year of the study on reproduction.

**Results:**

Statistical analysis showed that stallion breed was a significant influencing factor for the number of pregnant barren mares (Odds ratio [OR]=1.72; p=0.03; 95% confidence interval [CI]=1.05, 2.84) seasonal pregnancy rate (OR=1.40; p<0.001; 95% CI=1.29, 1.53). Additionally, the reproductive performance of the Barb stallion was superior to that of the Thoroughbred stallion. With regard to the significant influence of stallion’s age of >5 years on the number of pregnant foaling mares and seasonal pregnancy rate, significant interactions were observed between the stallion’s breed and age, and the pregnancy rate per cycle: in the Barb breed, the pregnancy rate per cycle increased with the age of the stallion, while in the Thoroughbred, it decreased with age. Moreover, a significant effect of the year of the study on the pregnancy rate per cycle and number of pregnant foaling mares was observed. In contrast, the number of cycles and embryonic mortality rate were not influenced by the breed and age of the stallion, or the year of the study.

**Conclusion:**

The Algerian Barb horse attained a similar level of fertility compared with that of the Arabian Purebred and Thoroughbred stallions depending on its age and reproductive performance.

## Introduction

Barb horse is the representative breed of equines of North Africa (Algeria), and has an important place in the history, culture, and tradition of the society. It is generally used in the Fantasia (traditional exhibition of horseback riding in the Maghreb), as well as in equestrian sports (endurance and jumping). In Algeria, the Barb is considered an autochthonous equine breed, with genetical relationship to the Arabian horse [1,2]. Currently, the importance of the Barb horse extends to other countries worldwide notably those in Europe (Spain, Italy, Malta, England, and France etc.) and sub-Saharan Africa (Madagascar, Mali, Senegal, and South Africa etc.) [3] Due to its excellent learning ability through characteristics of docile nature and highly developed faculties of assimilation and understanding [4]. However, the Algerian Barb population amounts to 100.000 horses (10.000 Barb and 90.000 Arab-Barb) [3,5].

To the best of our knowledge, several studies focused on genetic characterization and morphometry of Barb horses are reported in the literature [2,3], but, unlike studies on the Arabian Purebred and Thoroughbred worldwide, studies on the reproductive performance of the Barb horses in Algeria are lacking. The influencing factors of the breeding performance of horses are generally categorized as explanatory variables related to the stallion, mare, and management practices of animal husbandry [6].

In our study, we included the age and breed of the stallion and year of the study as variables in the analysis of reproductive parameters such as the number of operated cycles, number of pregnant mares by category (barren, maidens, and foaling), conception rate per cycle, seasonal pregnancy rate, and embryonic mortality rate. The study aimed to assess the influence of and interaction(s) among the different factors that may affect the fertility of the Arabian Purebred, Thoroughbred, and Barb horses.

We conducted a multivariate analysis of the fertility data obtained in stallions during ten consecutive mating seasons (2003-2012) at Chaouchaoua stud farm, Tiaret, Algeria, the location considered as the natural cradle of the Barb horse.

## Materials and Methods

### Ethical approval

This study was approved by the scientific council of High National Veterinary School, Algiers, Algeria.

### Animals and place of study

A retrospective study was conducted with the fertility records of the stallions at the Chaouchaoua National stud farm of Tiaret (Algeria), during ten consecutive breeding seasons (2003-2012). In Algeria, the season of administrative mating begins on February 15 and ends on June 15 of each year. In our study, a total of 168 stallions of different breeds (Barb, Arabian Barb, Arabian Purebred, and Thoroughbred), aged between 4 and 30 years old, and who served a large number of mares of corresponding breed (1592 cycles) were included. Mares were categorized into three types according to their physiological status at the beginning of the season: Barren mares considered as problematic with pathological endometritis, embryonic mortality, or abortion; foaling mares, arrived at the stud with a foal, and hence with reproductive history known; maidens, never bred, young or sporty.

### Detection of heat and mating

In mares in estrus (heat), daily trans-rectal ultrasound examination was carried out to monitor the follicular growth, and thereby, determine the time closer to ovulation and minimize the use of stallions avoidance of over-use of stallions). In case abnormal changes detected in the genital tract, rapid treatment was administered. Mating was conducted daily after a follicle diameter of >35 mm was attained until the time-point of ovulation, at an average of one to three matings per cycle and two matings per day per stallion (except for artificial insemination with fresh semen in case of overburden of the stallion), and diagnosis of pregnancy was made on post-ovulation day 14.

### Reproduction performance parameters

Several reproductive parameters were analyzed, such as the number of operated cycles (corresponding to the use of the stallion, total number of cycles of the mares in the reproduction challenge divided by the number of gravid mares). At the end of the season, pregnant mares were divided as barren, maiden, or foaling. Conception rate per cycle (the percentage of the number of mares that conceived divided by the total number of impregnated mares) was considered as the most sensitive criterion for assessing the stallion’s fertility, which reflects the actual capacity to initiate gestation. In addition, the embryonic death rate (early or late) and seasonal pregnancy rate were evaluated according to the stallion breed: Barb (Barb and Arabian Barb) and others (Arabian Purebred and Thoroughbred).

### Statistical analysis

For data collected in this study, descriptive statistical analysis was used, and continuous variables were presented as mean and standard deviation, while the categorical variables as absolute and percentage frequencies.

To study the impact of the characteristics of the stallion, including the breed (Barb *vs*. other breeds), age, and year of the study (from 2003 to 2012) on the reproductive performance indicators of mares, a multivariable generalized linear model was used.

To analyze reproductive performance in terms of the number of cycles operated, a linear regression model was used Results of the model were reported as the mean difference in the number of cycles operated with associated 95% Confidence Interval (CI) and p-value.


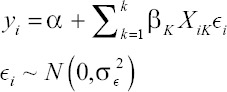


Where for the *i*^th^ observation, *γ_i_* is the number of cycles operated, *α* is the intercept of the model, *X_ik_* is a vector of *k* covariates (breed, age, and year), *β_k_* is the associated coefficient, and *ϵ_i_* is the error term.

To assess the impact of the characteristics of the stallion (breed and age) and year of the study on the reproductive performance indicators including the rate of gravid barren mares, pregnant foaling mares, and gravid maiden mares, conception rate per cycle, seasonal pregnancy rate, and early-embryonic death rate, logistic regression models were used For each of the indicators of reproductive performance, the results of the regression logistic model were presented as Odds Ratio (OR) of the dependent variable with associated 95% CI and p-value.


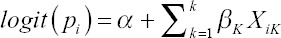


Where for the *i*^th^ observation, *p_i_* is the probability or rate of the respective reproductive performance indicator, *α* is the intercept of the model, *X_ik_* is a vector of *k* covariates (breed, age, and year), and *β_k_* is the associated coefficient.

For both these analyses, a model selection procedure consisting of two steps was carried out:

- Step 1, univariate analysis: The relationship of the explanatory variable of breed, age, and the year of study with each reproductive performance indicator was tested sequentially, using the likelihood ratio test. Variables with p<0.25 in univariate analysis at 95% confidence level were submitted to step 2, i.e., multivariate analysis [7]. The linearity effect of the continuous variable of age was checked for each model using the likelihood *ratio test* [8]; if the effect of age was non-linear, the variable was transformed into a categorical variable (<5 years, 5-10 years, and >10 years).
- Step 2, multivariate analysis: The best model to describe the reproductive performance data were selected using a forward-stepwise variable selection procedure (starting with an empty model) with the Akaike information criterion (AIC) [9]; the model with the smallest AIC value was chosen. All second-order interactions between covariates were tested in the final model.

The overall goodness-of-fit of the selected models was tested using Pearson Chi-square statistic [10].

Statistical analyses were performed using the R (version 3.4.0) software (R Development Core Team [11].

## Results

### Descriptive analysis

#### Reproductive performance indicators

In this study, the mean number of cycles operated was 9.5±9.4 (total cycles operated, 1592 in 1202 mares served), and the number of barren mares was 55.7±42.6%; foaling mares, 79.5±29.3%; and maiden mares, 9.9±36.3% (Table-1).

**Table 1:** Results of descriptive analysis of the reproductive performance indicators and characteristics of the stallion (sample n=168).

Variable	Descriptive indicator	Value
Reproductive performance indicators		
Number of cycles operated (n)	mean (SD)	9.5 (9.4)
Gravid barren mares rate (%)	mean (SD)	55.7 (42.6)
Pregnant foaling mares rate (%)	mean (SD)	79.5 (29.3)
Gravid maiden mares rate (%)	mean (SD)	79.9 (36.3)
Conception rate per cycle (%)	mean (SD)	66.3 (27.5)
Seasonal pregnancy rate (%)	mean (SD)	74 (29.4)
Embryonic mortality rate (%)	n (%)	10.2 (21.4)
Characteristics of the stallion		
Breed		
Barb	n (%)	44 (26.8)
Others	n (%)	124 (73.8)
Age		
<5years	n (%)	6 (3.5)
5-10years	n (%)	57 (33.9)
>10years	n (%)	105 (62.5)
Year of study	n (%)	
2003	n (%)	10 (5.9)
2004	n (%)	13 (7.7)
2005	n (%)	10 (5.9)
2006	n (%)	14 (8.3)
2007	n (%)	16 (9.5)
2008	n (%)	19 (11.4)
2009	n (%)	19 (11.4)
2010	n (%)	18 (10.7)
2011	n (%)	28 (16.7)
2012	n (%)	21 (12.5)

SD=Standard deviation

The early-embryonic death rate was 10.2±21.4%, and the conception rate per cycle and seasonal pregnancy rate were 66.3%±27.5% and 74%±29.4%, respectively.

#### Characteristics of the stallion

In this study, a total of 168 stallions were examined, of which 26.8% (n=44) were Barb and 73.8% (n=124) were Arabian Purebred and Thoroughbred. In addition, 3.5% (n=6) were aged <5 years, 33.9% (n=57) were aged 5–10 years, and 62.5% (n=105) were aged >10 years (Table-1).

#### Year of study

In this work, data for ten consecutive breeding seasons (2003-2012) were collected. The number of stallion per season ranged from 10 to 28 stallions (Table-1).

### Multivariable model

In the results of multivariate analysis, no significant effects of the breed, age of the stallion, and year of the study on the number of cycles operated, number of gravid maiden mares, and embryonic mortality rate were observed.

#### Rate of barren mares become pregnant

Significant effect by breed is shown in Table-2. Barren mares bred by a Barb stallion were more likely to be pregnant than those bred by a stallion of other breed of Arabian Purebred or Thoroughbred (OR=1.72; p=0.03; 95% CI=1.05, 2.84).

**Table 2:** Effect of the breed of the stallion on the number of barren mares become pregnant.

Variable	Category	Gravid barren mares rate (%) mean (SD)	Univariate analysis		Multivariateanalysis (final model)	
	
			Crude OR (95% CI)	p-value	Adjusted OR (95% CI)	p-value
Breedz	Others	53.1 (45.1)	Reference	0.03	Reference	/
	Barb	61.6 (36.3)	1.72 (1.05-2.84)		1.72 (1.05-2.84)	0.03
Age	<5years	42.8 (50.8)	Reference	0.61	/	/
	5-10years	55.5 (40)	1.30 (0.37-4.52)		/	/
	>10years	56.5 (43.8)	1.59 (0.48-5.27)		/	/
Year of study	2003	37 (41)	Reference	0.06	/	/
	2004	85.7 (37.8)	13.92 (2.31-270)		/	/
	2005	42.5 (46)	1.08 (0.28-4.19)		/	/
	2006	50 (47.1)	0.74 (0.22-2.46)		/	/
	2007	55.5 (48.6)	1.39 (0.49-4.03)		/	/
	2008	64.9 (38.4)	1.77 (0.62-5.15)		/	/
	2009	66 (37.3)	1.52 (0.56-4.22)		/	/
	2010	53.1 (44.3)	1.30 (0.43-4)		/	/
	2011	39.9 (44.7)	0.83 (0.30-2.26)		/	/
	2012	64 (38.3)	1.65 (0.61-4.56)		/	/

SD=Standard deviation, OR=Odds ratio, CI=Confidence interval

#### Number of foaling pregnant mares

Irrespective of breed, a significant effect of the age of the stallion was observed. Foaling mares bred by a stallion of >5 years-old were more likely to be pregnant than those bred by a stallion <5 years-old (Table-3).

**Table 3:** Influence of the age of the stallion and the year of the study on the number of foaling pregnant mares.

Variable	Category	Pregnant foaling mares rate (%) mean (SD)	Univariate analysis		Multivariateanalysis (final model)	
	
			Crude OR (95% CI)	p-value	Adjusted OR (95% CI)	p-value
Breed	Others	77.4 (32.1)	Reference	0.47	/	/
	Barb	85.4 (18.6)	1.17 (0.76-1.83)		/	/
Age	<5years	76.4 (34.2)	Reference	0.11	Reference	/
	5-10years	78.2 (32.7)	2.75 (0.88-8.04)		3.26 (1-10.08)	0.04
	>10years	80.3 (27.5)	3.17 (1.04-9.02)		3.73 (1.17-11.16)	0.02
Year of study	2003	76.4 (30.8)	Reference	<0.01	Reference	/
	2004	89.6 (16.3)	1.6 (0.53-4.71)		1.52 (0.53-4.71)	0.45
	2005	93.6 (10.8)	1.8 (0.65-5.13)		1.77 (0.65-5.13)	0.27
	2006	62.1 (35)	0.29 (0.12-0.61)		0.28 (0.12-0.61)	<0.01
	2007	78 (31)	1.02 (0.27-1.72)		0.69 (0.27-1.72)	0.42
	2008	84 (27.1)	1.23 (0.44-3.29)		1.19 (0.44-3.29)	0.74
	2009	87.9 (25.7)	1.42 (0.53-3.87)		1.4 (0.53-3.87)	0.5
	2010	77.3 (27.9)	0.81 (0.33-1.83)		0.79 (0.33-1.83)	0.58
	2011	72.6 (33.1)	0.7 (0.33-1.58)		0.74 (0.33-1.58)	0.45
	2012	81.3 (32.7)	1.34 (0.6-3.38)		1.44 (0.6-3.38)	0.4

SD=Standard deviation, OR=Odds ratio, CI=Confidence interval

Significant differences in the conception rate of foaling mares according to year of the study were obtained; particularly, a significantly lower rate was obtained in 2006 than 2003 (OR=0.28; p<0.001; 95% CI=0.12, 0.61), and no significant difference in the conception rates at the other years versus that at 2003 were obtained (Table-3).

#### Conception rate per cycle

In the analysis result, significant effect of the year of the study on the conception rate per cycle was observed; higher conception rate compared to that at 2003 was obtained for the years of 2004, 2005, 2007, 2008, 2009, and 2012; whereas, for the remaining years of 2006, 2010, and 2011, no significant difference in the conception rate of the mares was obtained (Table-4).

**Table 4:** Influence of breed, age of stallion, and year of study on conception rate per cycle.

Variable	Category	Conception rate per cycle (%) mean (SD)	Univariate analysis		Multivariateanalysis (final model)	
	
			Crude OR (95% CI)	p-value	Adjusted OR (95% CI)	p-value
Breed	Others	65.7 (30.1)	Reference	<0.01	Reference	/
	Barb	68.3 (18.3)	1.13 (1.05-1.21)		0.25 (0.16-0.4)	<0.001
Age	<5years	65.5 (20.3)	Reference	<0.001	Reference	
	5-10years	70.8 (29.5)	1.28 (1.07-1.52)		0.41 (0.26-0.62)	<0.001
	>10years	64 (26.6)	0.93 (0.78-1.11)		0.3 (0.19-0.45)	<0.001
Breed×Age	Barb and 5-10years	72.3 (19.8)	6.84 (4.07-10.7)	<0.001	4.75 (2.97-7.87)	<0.001
	Barb and>10years	67.9 (17.7)	7.33 (4.64-11.9)		5.12 (3.22-8.41)	<0.001
Year of study	2003	55.9 (19.6)	Reference	<0.001	Reference	/
	2004	70.3 (27.1)	1.87 (1.56-2.21)		1.85 (1.56-2.21)	<0.001
	2005	82.4 (17.2)	3.69 (2.68-4.06)		3.3 (2.68-4.06)	<0.001
	2006	60.8 (35.9)	1.22 (0.95-1.33)		1.13 (0.95-1.33)	0.16
	2007	64.7 (29.1)	1.45 (1.19-1.65)		1.4 (1.19-1.65)	<0.001
	2008	77.3 (27.1)	2.69 (2.09-2.92)		2.47 (2.09-2.92)	<0.001
	2009	72.7 (25.8)	2.1 (1.64-2.28)		1.93 (1.64-2.28)	<0.001
	2010	57.3 (21.7)	1.06 (0.83-1.14)		0.98 (0.83-1.14)	0.76
	2011	60.5 (29.5)	1.21 (0.95-1.29)		1.11 (0.95-1.29)	0.18
	2012	66.2 (27.4)	1.54 (1.21-1.65)		1.41 (1.21-1.65)	<0.001

SD=Standard deviation, OR=Odds ratio, CI=Confidence interval

Significant interaction between the age and breed of the stallion and the conception rate of mares was noted. In mares bred by a Thoroughbred stallion, there was a decline in the conception rate with increasing age of the stallion. Mares bred by stallions between 5 and 10 years of age had an OR of conception rate of 0.41 (p<0.001; 95% CI=0.26, 0.61), and mares bred by stallions older than 10 years had an OR of conception rate of 0.30 (p<0.001; 95% CI=0.19, 0.45) compared to mares bred by stallions younger than 5 years.

However, in mares bred by a Barb stallion, there was an increasing trend of the conception rate with increasing age of the stallion. Mares bred by stallions between 5 and 10 years-old had an OR of conception rate of 4.75 (p<0.001; 95% CI=2.97, 7.87) and mares bred by stallions older than 10 years had an OR of conception rate of 5.12 (p<0.001; 95% CI=3.22, 8.41) compared to mares bred by stallions younger than 5 years. Regarding the effect of the stallion’s breed, in the model results, the conception rate of mares bred by Barb stallions older than 5 years was significantly higher than that of mares bred by Thoroughbred stallions older than 5 years (Table-4).

#### Seasonal pregnancy rate

Significant effect of the year of the study on the seasonal pregnancy rate was obtained (Table-5). Higher seasonal pregnancy rate of mares than that in 2003 was obtained for the years of 2004, 2005, 2008, 2009, and 2012, and a lower pregnancy rate than that in 2003 was obtained for 2006; whereas for the remaining years of 2007, 2010, and 2011, no significant difference in the pregnancy rate of mares was obtained.

**Table 5:** Influence of breed, age of stallion, and year of study on seasonal pregnancy rate.

Variable	Category	Seasonal pregnancy rate (%) mean (SD)	Univariate analysis		Multivariateanalysis (final model)	
	
			Crude OR (95% CI)	p-value	Adjusted OR (95% CI)	p-value
Breed	Others	73 (31.6)	Reference	<0.001	Reference	/
	Barb	76.9 (22.5)	1.23 (1.14-1.34)		1.4 (1.29-1.53)	<0.001
Age	<5years	66 (32)	Reference	<0.001	Reference	/
	5-10years	75.1 (30.7)	1.55 (1.30-1.86)		1.95 (1.61-2.36)	<0.001
	>10years	73.8 (28.8)	1.45 (1.22-1.73)		1.83 (1.52-2.21)	<0.001
Year of study	2003	66.3 (20)	Reference	<0.001	Reference	/
	2004	84.9 (23.9)	2.86 (2.25-3.38)		2.75 (2.25-3.38)	<0.001
	2005	88.9 (11.6)	4.07 (2.91-4.69)		3.68 (2.91-4.69)	<0.001
	2006	53.1 (35.5)	0.57 (0.45-0.63)		0.53 (0.45-0.63)	<0.001
	2007	69.4 (33.8)	1.15 (0.92-1.29)		1.09 (0.92-1.29)	0.32
	2008	84.3 (24.3)	2.72 (2.26-3.24)		2.71 (2.26-3.24)	<0.001
	2009	84.1 (24.4)	2.68 (2.09-3)		2.5 (2.09-3)	<0.001
	2010	69.7 (27.3)	1.17 (0.89-1.24)		1.05 (0.89-1.24)	0.55
	2011	66.2 (32.6)	0.99 (0.78-1.06)		0.91 (0.78-1.06)	0.21
	2012	76.9 (31.5)	1.69 (1.4-1.95)		1.65 (1.4-1.95)	<0.001

SD=Standard deviation, OR=Odds ratio, CI=Confidence interval

With regard to the effect of the stallion’s breed on the seasonal pregnancy rate, mares bred by a Barb stallion had a significantly higher gestation rate than mares bred by Arabian Purebred stallion or Thoroughbred (OR=1.40; p<0.001; 95% CI=1.29,1.53) (Table-5).

Irrespective of the breed of the stallion, an increasing trend of the gestation rate of mares with increasing age of the stallion used for breeding, was observed: OR of the age group of 5-10 years and >10 years of 1.95 (p<0.001; 95% CI=1.61, 2.36) and 1.83 (p<0.001; 95% CI=1.52, 2.21), respectively compared to the <5 years’ age group, were obtained (Table-5).

## Discussion

We observed that the type of breed had a significant effect on the number of pregnant mares that were barren the previous year, but not on that of foaling and maiden mares. Mares that underwent mating with a Barb stallion were more likely to be gravid than those that underwent mating with a stallion of another breed. Barb stallions were more fertile than Thoroughbred and Arabian Purebred stallions, even when bred with mares with reproductive problems or low fertility (subfertile) in the harem [12]. The breed and age of the stallion and year of the study were not significant influencing factors in a total of 1592 operated cycles. Mares with extended conception due to embryonic mortality, abortion, or not having been fertilized by another stallion showed variation in the seasonal pregnancy rate due to a decrease in the number of operated cycles and fertility of the stallion; therefore, breeders should have information on the percentage of each category of pregnant mares in the harem. A stallion with good fertility at the end of the season or when bred with mares with good breeding potentials such as foaling and maiden mares, may show a low fertility rate when bred with mares with reproductive dysfunctions such as barren mares [12].

In addition, our findings demonstrated that the breed had a significant influence on the per cycle pregnancy rate (68.3 ±18.3% for the Barb vs. 65.7 ±30.1% for the Thoroughbred and Arabian purebred) and on the seasonal pregnancy rate (76.9±22.5% for the Barb vs. 73%±31.6% for the Thoroughbred and Arabian Purebred).

Gibb *et al.*, [13] reported that the conception rate per cycle of Thoroughbred stallions was extremely variable, ranging from 35% to 90%; our results of a conception rate of 65.7% corroborated with those of a study on Thoroughbreds in Australia (54%–64%) [14], that in Pakistan (62%) [15] (in the 1^st^ cycle), that in UK (63%) [16], that in the USA (65%) [17], that in Australia (68.8%) [18], and that in Ireland (68%) [19].

However, our conception rate result was higher than the value of 53.6% reported for Thoroughbreds by Hanlon *et al.*, [20], and lower than 80% and 84% reported for the Arabian Purebred by Demirci, [21], and Benhajali *et* *al*. [22].

The differences in the fertility of the two breeds could be explained by the genetic variability and its relation to the semen characteristics [23,24]. Dowsett et Knott, [23] reported that the Arabian Purebred demonstrated higher total harvested volume (with reduced gel fraction), and high sperm concentration (with high rate of viability), as compared to the Thoroughbred [23].

In our study, the older stallions obtained a lower conception rate, which could be explained by the finding of significant decline in the quality of semen by age (>15 years) in Arabian stallions in Tunisia [25].

With regard to seasonal pregnancy rates, our findings were similar to those of a study in the USA [26], but lower than 85% and 89% obtained by Hanlon *et al*. [20] and Bosh *et al*. [17], respectively.

With regard to seasonal pregnancy in the Arabian Purebred, studies in Turkey [27] and Algeria [28] reported a rate of 69% and 62% respectively. In our study, the conception rate and seasonal pregnancy rate were significantly higher in mares mated with Barb stallions than those mated with stallions of other breeds of Arabian Purebred or Thoroughbred. The high fertility of the Algerian Barb could be explained in part by better adaptation and endurance under natural environment in its native setting.

Additionally, we found a significant age-breed interaction on the conception rate per cycle: the conception rate of mares was increased with older Barb stallions while decreased with Arabian Purebred and Thoroughbred stallions. The age and year of the study had a significant effect independent of breed, on the number of foaling pregnant mares in the harem. In this regard, Langlois [29] reported that the young stallion was able to fertilize ova at 2 years of age, which enabled fertility evaluation.

Different studies have indicated the influence of age of the stallion on sperm production and quality [23,29-31], especially in young and old stallions. The age effect may be due to differences in daily sperm production and ejaculation, which are related to factors such as immature spermatogenesis in foals, and testicular degeneration due to aging and aberrant epidermal function [23,32].

Most sperm characteristics (total volume, volume of gel, and total number of spermatozoid) are influenced by the stallion’s age, but are not considered as influencing factors of fertility except in very young individuals of <3 years-old or elderly individuals of > 14 years-old [30].

With regard to the influence of the stallion’s age on the fertility of foaling mares and conception rate per cycle, our results were similar to the findings on sexual maturity in horses over the age of 5 years by Ortega-Ferrusola *et al.*, [33]. Dowsett and Knott, [23] included foals under 3 years of age and reported successful harvesting of a small volume of sperm with low concentration of spermatozoid, reduced total spermatozoid number, and higher percentage of abnormal spermatozoid.

Studies have indicated the poor quality of sperm in older stallions of the age range of 11 years and older, which suggests a critical age for the decline in sperm quality; nevertheless, some stallions may produce a higher number of defects due to defective epididymal maturation and spermatogenesis.

According to these phenomena, our findings indicated that the age of the stallion was an influencing factor for the seasonal pregnancy rate (OR=1.95; CI=1.61, 2.36 for 5-10 years old; OR=1.83; CI=1.52, 2.21 for >10 years old *vs*. OR=1.40; CI=1.29, 1.53 for <5 years old; p<0.001, 95% CI). Young horses are contraindicated for breeding or may be used only if the testicular size and libido are adequate, and at once-a-day frequency [34], and older horses may be used but under regular control of the semen quality [23].

The conception rate in foaling mares, conception rate per cycle, and seasonal pregnancy rate were significantly affected by the year of the study, which may be explained by differences in the breeding practice (feeding) and reproductive management (gynecological and ultrasound monitoring), or related to the environmental factors (climate, temperature, lighting, and season) [27,31,35].

Despite the limitation of our study of single-center design at one stud (Haras of Tiaret), we reported a conception rate per cycle of 66.3±27.5%, which is similar to that reported by Allen *et al.*, [16] in UK (1011 mares) and Bosh *et al*. [17] in USA (2321 mares); however, we obtained a much lower seasonal pregnancy rate of 74±29.4% compared with that obtained in previous studies.

Our results are relevant to the racing industry that emphasizes the requirements and financial means of the owners, and optimal conditions of reproductive management such as close gynecological monitoring.

In our study, the embryonic mortality rate (early or late) of 10.2% was not affected by the age and breed of the stallion, or year of the study, and the value was higher than those of Allen *et al*. [16] (7.2-8% between 15 and 42 days) and Nath *et al*. [18] (7.1% for the Thoroughbred, and 7.5% for the Standardbred), but lower than those of 15%, 12.5%, and 15.3% of Lane *et al*. [19], Hemberg *et al*. [36] and Schnobrich *et al*. [37], respectively.

The age of mares was considered as one of the etiological factors of embryonic mortality, which could explain the low pregnancy rate of older mares caused by the poor quality of oocytes (low viability of the embryos), alteration of fertilization, unfavorable environment of the oviduct, progressive degenerative changes of the endometrium, and susceptibility to infection in older mares [36,37].

Other intrinsic and extrinsic factors that negatively influence both the mother and the embryo include luteal insufficiency, endometritis, intrauterine fixation site of the embryonic vesicle, climate, season, stress, diet, and chromosomal abnormalities [38].

To improve the reproductive efficiency of mares and reduce the rate of embryonic mortality, in all cases, clinicians should conduct careful examination of the genital tract, such as cytological examinations of the endometrium [36], as well prophylactic treatments of vaccination and deworming, and ensure sanitary measures such as treatment of uterine infections and vulvar conformation, and proper nutrition to maintain a good body condition of the mares [36].

## Conclusion

Our study demonstrated that the stallion’s age was an important consideration in breeding practice. The breed and age of the stallion, and year of the study were significant influencing factors for the conception rate per cycle and seasonal pregnancy rate in normal mares or those with reproductive dysfunction (foaling or barren types). The Barb achieved better reproductive performance in its native habitat, and it can significantly compete in terms of fertility compared to Thoroughbred and Arabian Purebred. Our study highlighted that Barb stallion at the optimum age of >5 years and >10 years had better performance and higher ability to attain conception even in mares with reproductive dysfunction.

## Authors’Contributions

NA and AN designed the database of fertility data for the Stallion of Chaouchaoua Stud Farm. MAB conducted the statistical analysis. NA drafted the manuscript under the guidance of RK and AKM. All authors read and approved the final version of the manuscript.
